# Metabolic Fuel for Epigenetic: Nuclear Production Meets Local Consumption

**DOI:** 10.3389/fgene.2021.768996

**Published:** 2021-11-03

**Authors:** Ruben Boon

**Affiliations:** ^1^ The Massachusetts General Hospital Cancer Center, Harvard Medical School, Boston, MA, United States; ^2^ The Broad Institute of Harvard and MIT, Cambridge, MA, United States; ^3^ Laboratory for Functional Epigenetics, Department of Human Genetics, KU Leuven, Leuven, Belgium

**Keywords:** nuclear metabolism, epigenetics, liquid-liquid phase separation (LLPS), compartmentalization, chromatin

## Abstract

Epigenetic modifications are responsible for finetuning gene expression profiles to the needs of cells, tissues, and organisms. To rapidly respond to environmental changes, the activity of chromatin modifiers critically depends on the concentration of a handful of metabolites that act as substrates and co-factors. In this way, these enzymes act as metabolic sensors that directly link gene expression to metabolic states. Although metabolites can easily diffuse through the nuclear pore, molecular mechanisms must be in place to regulate epigenetic marker deposition in specific nuclear subdomains or even on single loci. In this review, I explore the possible subcellular sites of metabolite production that influence the epigenome. From the relationship between cytoplasmic metabolism and nuclear metabolite deposition, I converse to the description of a compartmentalized nuclear metabolism. Last, I elaborate on the possibility of metabolic enzymes to operate in phase-separated nuclear microdomains formed by multienzyme and chromatin-bound protein complexes.

## Introduction

In the last 2 decades, the field of epigenomics has identified an immense variety of chromatin modifiers that collaborate to establish the epigenetic code. Evidently, the deposition of epigenetic marks is determined through genetic regulation of the modifiers (Cavalli and Heard, 2019; Zhao et al., 2021). However, cellular metabolism has now emerged as a second layer of regulation. Indeed, whereas chromatin writers, readers, and erasers establish the marks, their activity critically depends on a handful of metabolites. In this way, chromatin modifiers act as sensors for metabolite levels and directly link mark deposition or removal with the metabolic state of the cell, organ, or organism (Boon et al., 2020a; Haws et al., 2020a; Dai et al., 2020).

Cellular metabolism is the cluster of enzymatic reactions that occur in cells to transform nutrients such as glucose, fatty acids, amino acids, and vitamins into cellular components, energy, and reducing power. As metabolites can freely diffuse through the relatively large nuclear pores, the nucleus has been proposed to be in metabolic homeostasis with the cytoplasm (Wente and Rout, 2010; Cambronne et al., 2016). Yet, fluctuations in cytoplasmic metabolites should not lead to random epigenetic changes in the nucleus. Furthermore, both the cytoplasm and nucleus represent highly crowded environments where molecular bindings and collisions severely limit the diffusion of molecules (Ellis, 2001; Richter et al., 2008). It is therefore unlikely that passive diffusion of metabolites from the cytoplasm into the nucleus forms the basis of the metabolism-epigenetic link.

In this review, I explore where the metabolic fuel of epigenetic modifiers might be produced. First, I show a correlation between epigenetic marks and cytoplasmic metabolism. Second, I review studies indicating a distinct nuclear metabolism based on the import of metabolic enzymes. Last, I elaborate on observations indicating further subnuclear compartmentalization based on protein complexation and phase separation.

## The Epigenome Is a Sensor Linking Metabolome and Transcriptome

Chromatin modifiers can chemically alter histones, DNA, and RNA to change chromatin structure, adjust the accessibility of genetic elements, and determine the stability of transcripts (Allis and Jenuwein, 2016). Although historically methylation, acetylation, and phosphorylation have been described most extensively, novel epigenetic modifications of especially histones including lactylation (Zhang et al., 2019), β-N-acetylglucosaminylation (Fujiki et al., 2011) (O-GlycNACylation), and acylation (Sabari et al., 2017) are increasingly appreciated as major determinants of transcription. Notably, the deposition and removal of these marks are dependent on the production of metabolites that act as substrates, co-factors, and repressors of chromatin modifiers. As the concentration of metabolites can influence the activity of chromatin modifiers, these enzymes themselves act as metabolic sensors that translate changes in cellular metabolism to transcriptional reprogramming (Rabhi et al., 2017).

Indeed, metabolic pathways, and even single metabolites, change cellular behavior (Guijas et al., 2018). For instance, in pancreatic cancer, the expression of hundreds of genes involved in the p53 antitumor/differentiation response relies on the accumulation of a single metabolite; α-ketoglutarate (αKG) (Morris et al., 2019). In other systems, supplementation of αKG can improve cellular reprogramming (Carey et al., 2015) and accelerate the differentiation of primed pluripotent stem cells (TeSlaa et al., 2016), colon cancer (Tran et al., 2020), and epidermal stem cells (Baksh et al., 2020). αKG drives cell fate changes because it acts as a critical co-factor for histone and DNA demethylases. Accumulation thus leads to the opening up of chromatin structures and the expression of responsive genes that were previously silenced. In this regard, αKG-derived metabolites such as succinate, fumarate, and 2-hydroxyglutarate (2HG) have been identified as the first oncometabolites through inhibition of epigenetic demethylation and subsequent silencing of tumor surpressors (Shim et al., 2014; Sciacovelli and Frezza, 2016; Sulkowski et al., 2020). As summarized by Dai. Z et al. (Dai et al., 2020), an increasing collection of metabolites ranging from acetyl-CoA to S-adenosylmethionine (SAM) affect the activity of chromatin modifiers and directly translate metabolic states to transcriptional reprogramming through epigenetic modifications. Diet is therefore increasingly appreciated as an additional way to target the epigenome to influence inflammation (Wang et al., 2018), metabolic disease (Yuan et al., 2018), and neurogenesis defects (Benjamin et al., 2017). Furthermore, in the field of stem cell technology, metabolic engineering of cell culture media is now exploited to regulate cell fate and maintain stem cell pluripotency (Tsogtbaatar et al., 2020), or drive neural (Yanes et al., 2010), hepatic (Boon et al., 2020b), and hemapoietic (Ito et al., 2019) lineage specification.

Cellular metabolism is not only regulated by cell-intrinsic signaling pathways. Given that metabolic pathways consume nutrients, there is extensive cross-reactivity between cellular metabolism and the composition of the nutrient microenvironment (Elia and Haigis, 2021). For example, in the pyruvate-and proline-rich lung microenvironment, metastatic breast cancer cells preferentially catabolise these nutrients to sustain energy production and the production of extracellular matrix components (Elia et al., 2017; Elia et al., 2019). Additionally, lactate buildup in metabolically active and poorly vascularized non-small-cell lung cancers (NSCLCs), induces the use of this “waste” product as a substantial carbon source for fueling mitochondrial oxidative metabolism (Faubert et al., 2017). Last, nutrient depletion observed in most tumors leads to metabolic inactivation of immune cells. T cells are metabolically outcompeted by tumor cells in terms of glucose (Cascone et al., 2018), methionine (Bian et al., 2020), and arginine catabolism (Geiger et al., 2016).

The epigenome thus lies between the nutrient microenvironment and the regulation of cell fate. In this way, epigenetics might act as a metabolic antenna that probes cellular metabolism and translates environmental changes into transcriptional responses. Although the exact modifier was not identified, recently, a novel histone modification, histone lactylation, was indeed found to modulate wound healing and homeostasis of macrophages as a response to high levels of lactate as found in sepsis and hypoxia (Zhang et al., 2019). These findings also translate to the lactate-high tumor microenvironment. Here, histone lactylation drives ocular melanoma through the induction of YTH N6-Methyladenosine RNA Binding Protein 2 (YTHDF2) responsible for degrading N6-methylated RNA of the tumor supressors PER1 and TP53 (Yu et al., 2021). Histone lactylation indeed acts as a metabolic sensor. In non-small cell lung cancer, high intracellular lactate leads to the deposition of lactylation marks in the promoters of the glycolytic enzymes (Hexokinase 1 and pyruvate kinase-M), leading to a reduction in their expression, and a redirection of energy production from lactate-producing glycolysis to mitochondrial metabolism (Jiang et al., 2021). Furthermore, the activity of the histone acetylation reader BRD4, which is responsible for driving transcription of oncogenic programs such as those regulated by MYC, was found to depend on intracellular purine levels, thus balancing transcription with the availability of extracellular and salvaged purines (Li et al., 2021). As another example, the production of ketone bodies during fasting can induce histone (β-hydroxy) butyrylation, which in turn transcriptionally induces the starvation response (Xie et al., 2016). As elaborated on below, the epigenome as a metabolic sensor allows for rapid transcriptional adaptations to balance metabolism with cellular output.

## Histone Acetylation and SIRT6 as Sensors for Cellular Energy Metabolism

**FIGURE 1:**
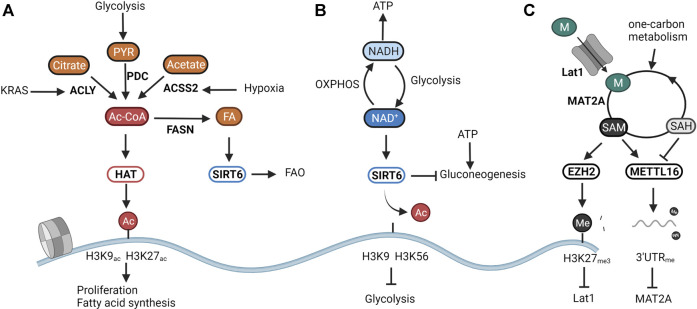
The epigenome senses cellular metabolism. Due to their enzymatic function, the activity of chromatin modifiers depends on the availability of certain metabolites. They can thus be considered metabolic sensors. **(A)** Histone acetyltransferases (HATs) respond to changes in central carbon metabolism by sensing intracellular levels of acetyl-CoA. Overflow of catabolism results in the induction of HAT activity and consequently in the increase of proliferation and storage of acetyl-CoA in fat reserves through Fatty Acid Synthase (FASN). **(B)** SIRT6 modulates glucose and fat metabolism as a response to the cellular energy state as represented by the NAD^+^/NADH ratio. High NAD^+^ availability induces mitochondrial metabolism responsible for generating NADH for energy production. A low NAD^+^/NADH ratio translates into the induction of glycolysis to regenerate NAD^+^. **(C)** Epigenetic methylation balances intracellular SAM levels needed for methylation reactions. SAM overflow induces the downregulation of the methionine transporter Lat1 through EZH2-mediated silencing. Low SAM levels are restored through the inability of the RNA methyltransferase METTL16 to methylate the RNA of the SAM producing enzymes MAT2A, resulting in increased translation and production. Created with BioRender.com.

The metabolic sensory properties of the epigenome are best illustrated through histone acetylation. The acetylation of histones by acetyltransferases (HATs) has been linked with active promoter and enhancer regions (Wang et al., 2008). Acetyl-CoA, the substrate of HATs, is a metabolite that lies at the heart of energy metabolism. It can be generated from pyruvate through the pyruvate dehydrogenase complex (PDC), from acetate through the Acyl-coenzyme A synthetase short-chain family member 2 (ACSS2), and from citrate through (ATP)-citrate lyase (ACLY). Acetyl-CoA thus links glucose, amino acid, and fatty acid metabolism to mitochondrial function and ATP production (Shi and Tu, 2015). Most HATs sense acetyl-CoA levels as intracellular levels of acetyl-CoA fluctuate around the Michaelis constant (Km)of these enzymes (Cai et al., 2011; Diehl and Muir, 2020). Wheeras some HATs. Might have evolved to maintain specific acetylation marks even under low acetyl-CoA availability, fluctuatins in acetyl-CoA levels due to inhibition of glycolysis directly affect almost half of all acetylated histones (Cluntun et al., 2015). As illustrated in Figure 1A, the activity of HATs directly links acetyl-CoA levels to transcriptional metabolic changes. In yeast, elevated levels of cellular acetyl-CoA lead to a global increase in acetylation of H3 and H4 lysine residues and activation of growth programs through the general control of amino acid synthesis protein 5-like 2 (GCN5) HAT (Cai et al., 2011; Shi and Tu, 2013). In accordance, oncogenic signals that arise during pancreatic tumorigenesis increase ATP-citrate lyase (ACLY) -dependent elevation of acetyl-CoA levels which in turn fuel histone acetylation in genetic loci linked to PDA progression (Carrer et al., 2019). Besides linking cellular proliferation with acetyl-CoA availability, HATs also seem to balance acetyl-CoA release from, and storage into fat reserves. For example, hypoxic conditions that increase intracellular acetyl-CoA levels through ACSS2-dependent acetate catabolism also induce acetylation of H3K9, H3K27, and H3K56 present in promoters of genes involved in fatty acid synthesis (Gao et al., 2016). Additionally, fat overload as observed in high-fat diets reduces the production of cellular acetyl-CoA, in turn decreasing histone acetylation and leading to a downregulation of fatty acid synthesis genes (Carrer et al., 2017). These examples thus clearly identify HATs as metabolic regulators that utilize acetyl-CoA levels to control proliferative and metabolic pathways (Figure 1A).

Besides the intracellular levels of acetyl-CoA, histone acetylation is further finetuned by deacetylation. A handful of studies have shown the ketone body d-β-hydroxybutyrate (βOHB) (Shimazu et al., 2013), lactate (Latham et al., 2012), and CoA derivatives (Vogelauer et al., 2012) to inhibit the activity of the classical histone deacetylases (HDAC1-10). Yet it is not known whether these act as metabolic sensors in physiological conditions. More evidence is available for highlighted the NAD^+^-dependent sirtuins (SIRT1-7)as textbook examples of metabolic sensors. NAD^+^ is a central metabolite that facilitates electron transfer derived from catabolic pathways. The ratio between NAD^+^ and its reduced form NADH thus represents cellular catabolic capacities and metabolic fitness. Whereas high NAD^+^ levels indicate low catabolic activity, low NAD^+/^NADH induces a shutdown of catabolism and regeneration of NAD Cantó et al., 2015. From all sirtuins, SIRT6 and SIRT7 are exclusively located in the nucleus. Whereas the poorly studied SIRT7 has been linked to DNA repair through deacetylation of H3K18ac (Barber et al., 2012), and to the activation of rRNA transcription through modulation of RNA polI (Ford et al., 2006), SIRT6 is an epigenetic regulator of glucose and fat metabolism through the removal of H3K9 and H3K56 acetylation (Chang et al., 2020). A shown in Figure 1B, when NAD^+^ levels are high, SIRT6 deacetylates H3K9 on the promoters of key glycolytic genes. This represses HIF-1α-dependent induction of glycolysis, and most importantly, of lactate dehydrogenase (LDH) (Zhong et al., 2010). In proliferating cancer cells, lactate secretion is responsible for regenerating NAD^+^ needed to maintain the glycolytic flux fueling cellular proliferation (Warburg effect) (Luengo et al., 2021). In normal cells, SIRT6 balances NAD^+^ levels by diverting glucose-derived carbons towards the mitochondrial TCA for the conversion of NAD^+^ to NADH and subsequent ATP production. Reduction of SIRT6 activity through low NAD^+^ availability or in an oncogenic setting results in a derepression of glycolytic genes, and, in the latter case, the acquisition of a metabolic profile driving uncontrolled proliferation (Sebastián et al., 2012; Choi et al., 2021). Especially in the liver, SIRT6 activity also determines the levels of gluconeogenesis. SIRT6 deacetylates and activates the acetyltransferase GCN5, resulting in an inhibition of PGC-1α-dependent gluconeogenesis. Furthermore, SIRT6 also deacetylates and induces nuclear exclusion of the gluconeogenic driver forkhead box protein O1 (FOXO1). As both reactions are dependent on NAD^+^, SIRT6 links gluconeogenic output and hepatic energy state through the NAD^+^/NADH ratio (Dominy et al., 2012; Zhang et al., 2014). Interestingly, the activity of SIRT6 was found to be amplified upon binding with free fatty acids (Feldman et al., 2013). As SIRT6 can also induce a switch from fatty acid synthesis to β-oxidation (FAO), its nuclear activity balances both glucose and fatty acid metabolism to NAD^+^ and fatty acid availability (Kim et al., 2010; Naiman et al., 2019) (Figure 1B).

## Histone and DNA Methylation as a Sensor for One-Carbon Metabolism

Whereas acetylation of histones is mainly linked to active transcription, methylation of histones and DNA is generally associated with inhibition of transcription and chromatin compaction. Depending on the methylated lysine residue, some methylation marks such as H3K4_me3_ and H3K36_me3_ are however linked with activating promoter activity and driving transcriptional elongation. Interestingly, as all methyltransferases (MTs) consume one molecule of the metabolite SAM and yield one molecule of the competitive inhibitor SAH, most MT’s seem to sense the SAM/SAH ratio. As shown in Figure 1C, this ratio in turn correlates with the availability of the SAM precursor methionine and with the cellular capacity to regenerate methionine from SAH by using one-carbon units. For instance, dietary restriction of methionine in animal studies significantly impacts both levels of SAMe as well as general DNA and histone methylation patterns (Mattocks et al., 2017; Miousse et al., 2017). Methionine restriction in both mice and humans also leads to changes in H3K4_me3_ deposition resulting in widespread metabolic reprograming (Mentch et al., 2015). In cancer models, this nutritional stress translates into a reduction in growth through decreased deposition of H3K4_me3_ on promoters of the cancer-associated genes AKT1, MYC, and MAPK (Mentch et al., 2015). In embryonic stem cells, a depleted one-carbon metabolism due to threonine depletion, or reduction of methionine availability results in low SAM levels, and a reduction of H3K4_me3_ in promoters of genes involved in proliferation and maintenance of stemness (Shyh-Chang et al., 2013; Shiraki et al., 2014).

Sensing the capacity to generate one-carbon units also acts as a prerequisite for the differentiation of T helper cells and macrophages. Here, only when nutrient availability allows for high SAM production, H3K4_me3_ or H3K36_me3_ marks are deposited in genes responsible for this transformation (Yu et al., 2019). For the macrophages, LPS-induced activation activates glucose, methionine, and one-carbon metabolism for increasing the SAM/SAH ratio. This leads to the deposition of H3K36_me3_ along the exons of IL-1β, facilitating the production of this interleukin. Along the same lines, in T helper cells, methionine restriction leads to a failure to induce the H3K4me3-dependent expression of genes determining cellular proliferation and T helper 17 (Th17) lineage specificity and pathogenicity (Roy et al., 2020).

As shown in Figure 1C, epigenetic methylation thus balances SAM levels to cellular needs and nutritional availability. In this regard, epigenetic modifiers were found to inherently balance SAM levels with its production through epigenetic methylation. When the production of SAM exceeds cellular needs, excess SAM is utilized by the HMT EZH2 for deposition of the silencing marker H3K27_me3_ on promoters of genes involved in the generation of SAM (Dann et al., 2015). Furthermore, when SAM levels fall below a certain threshold, the RNA MT METTL16 is no longer able to methylate the RNA of MAT2A, leading to its accumulation and increased production of SAM from methionine (Pendleton et al., 2017). Histone and DNA methylation then calibrate the expression of proliferative and metabolic genes to match nutritional availability (Figure 1C).

The evolving collection of histone modifications that are sensitive to fluctuations in metabolite levels was recently reviewed by Dai Z. et al. (Dai et al., 2020) It will be interesting to continue to teak out the connection between fluctuating metabolite levels and epigenetic responses to balance these. Yet fluctuations of metabolites often lead to very specific transcriptional responses of only a small set of (metabolic) genes (Cai et al., 2011; Dann et al., 2015; Yu et al., 2019). This thus raises the question of whether metabolite levels influence chromatin genome-wide or rather in a more locus-specific manner.

## Chromatin Acts as a Genome-Wide Metabolic Reservoir for Metabolites

Chromatin contains an enormous amount of proteins that are chemically modified with metabolic products. The number of modifications of histones, DNA, and RNA is so large that, especially for methylation and acetylation, epigenetic modifications represent a significant metabolic consumption for proliferating cells (Diehl and Muir, 2020). As each histone can take up to 400 molecules of SAM and 100 molecules of acetyl-CoA, the complete methylation and acetylation of chromatin require more than 1,000 and 100 fold the amounts of cellular SAM and acetyl-CoA, respectively (Martinez-Pastor et al., 2013; Ye and Tu, 2018). Histone methylation as a whole indeed consumes a large part of cellular SAM as blocking of H3K4, H3K36, and H3K79 methylation increases SAM levels in yeast more than 100 fold (Ye et al., 2017). Yet, the same study also showed yeast cells to be able to selectively demethylate subsets of histones. Indeed, upon SAM depletion, yeast cells selectively mobilize H3K36 and H3K79 methylation to sustain proliferation. Under these conditions, one-carbon units liberated by demethylating these histones fuel the generation of SAM. SAM, in turn, is then consumed in high amounts by the enzyme Phosphatidylethanolamine N-Methyltransferas (PEMT) responsible for generating the phospholipid phosphatidylcholine (PC). As PC is critical for forming lipid bilayers, demethylation of histones represents a mechanism to redirect resources from epigenetic storage towards a metabolic sink. (Ye et al., 2017). These histone marks are interesting as they are very abundant and found throughout the genome. H3K79 methylation is observed on 90% of all yeast histones and is responsible for shunting silencing factors towards silenced loci (van Leeuwen et al., 2002) and for silencing telomeres (Lacoste et al., 2002). Both marks have been found in gene bodies and mediate transcriptional elongation and termination (Bannister et al., 2005; Kizer et al., 2005), whereas H3K36 methylation also determines exons (Kolasinska-Zwierz et al., 2009). As small fluctuations in these marks were not found to severely change gene expression profiles in yeast, H3K36 and H3K79 could represent ideal nuclear methylation storages capable of taking up molecules of SAM when available, and releasing one-carbon units when production of SAM is compromised (Ye et al., 2017). A recent study has described a similar observation in mammalian cells. Upon methionine restriction, a variety of human cell lines was able to reduce H3K9_me3_ and H3K9_me2_ marks. The cells were able to mobilize these methylgroups without compromising the vast heterochromatin as the H3K9me1 mark was maintained to preserve silencing (Haws et al., 2020b). Mechanisms must thus be in place to selectively target subsets of histone marks that act as either transcriptional regulators of metabolic storage units.

It has been proposed that certain epigenetic marks are more sensitive to metabolite concentrations as the Km values of their respective writers and erasers differs (Mentch and Locasale, 2016). Interestingly, the Km values of MTs responsible for establishing H3K36, H3K79, and H3K9 methylation do not predict these marks to be the first to drop upon SAM starvation. Although ASH1L and SETD2 responsible for methylating H3K36 possess a relatively high Km value around 3 µM (Eram et al., 2015), the H3K79 MT DOTL1 still operates at 0.7 µM (Yu et al., 2012), whereas also KMT1B and KMT1C possess KM values of 0.74 and 1.8 µM of SAM, much lower than other epigenetic MTs (Mentch and Locasale, 2016). Signaling pathways must thus exist to selectively methylate or demethylate these marks, or even target specific locations. Indeed, in yeast under SAM depletion, Protein Phosphatase PP2A was found to probe SAM levels and to specifically induce the demethylation of H3K36 and H3K79 through activation of specific histone demethylases (Ye et al., 2019).

As mentioned before, metabolic overflow of acetyl-CoA induces the deposition of H3K9, H3K27, and H3K56 acetylation on genes involved in proliferation and fatty acid synthesis (Gao et al., 2016; Carrer et al., 2019). Cellular concentrations of acetyl-CoA seem to, in all conditions, far exceed the Michaelis constants of all human KATs. Transferase activity therefore might be dependent on the ratio between acetyl-CoA and the inhibitory free CoA, or even through the concentration of other acyl-CoA moities (Montgomery et al., 2015). Yet, even this would not explain why some genetic loci act as storage for acetylation and regulate transcriptional responses, while other are not. Maintenance of the epigenetic imprint as a whole is vital for the maintenance of cellular identity and function, and marker deposition or depletion should be targeted to specific genetic loci (Barrero et al., 2010). As described below, the metabolic fuel for epigenetics thus needs to be produced in a compartment near chromatin, and should even be limited to targeted loci.

## NAD^+^ Metabolism Is Compartmentalized Between the Nucleus and Cytoplasm

When looking at metabolic compartmentalization, several groups have proposed the existence of a separated nuclear metabolism that operates in contact with cytoplasmic metabolism (Sivanand et al., 2018; Boon et al., 2020a). Although metabolites are expected to freely diffuse through the relatively large nuclear pore (Wente and Rout, 2010; Cambronne et al., 2016), metabolic enzymes can selectively be imported or excluded from the nucleus.

**FIGURE 2:**
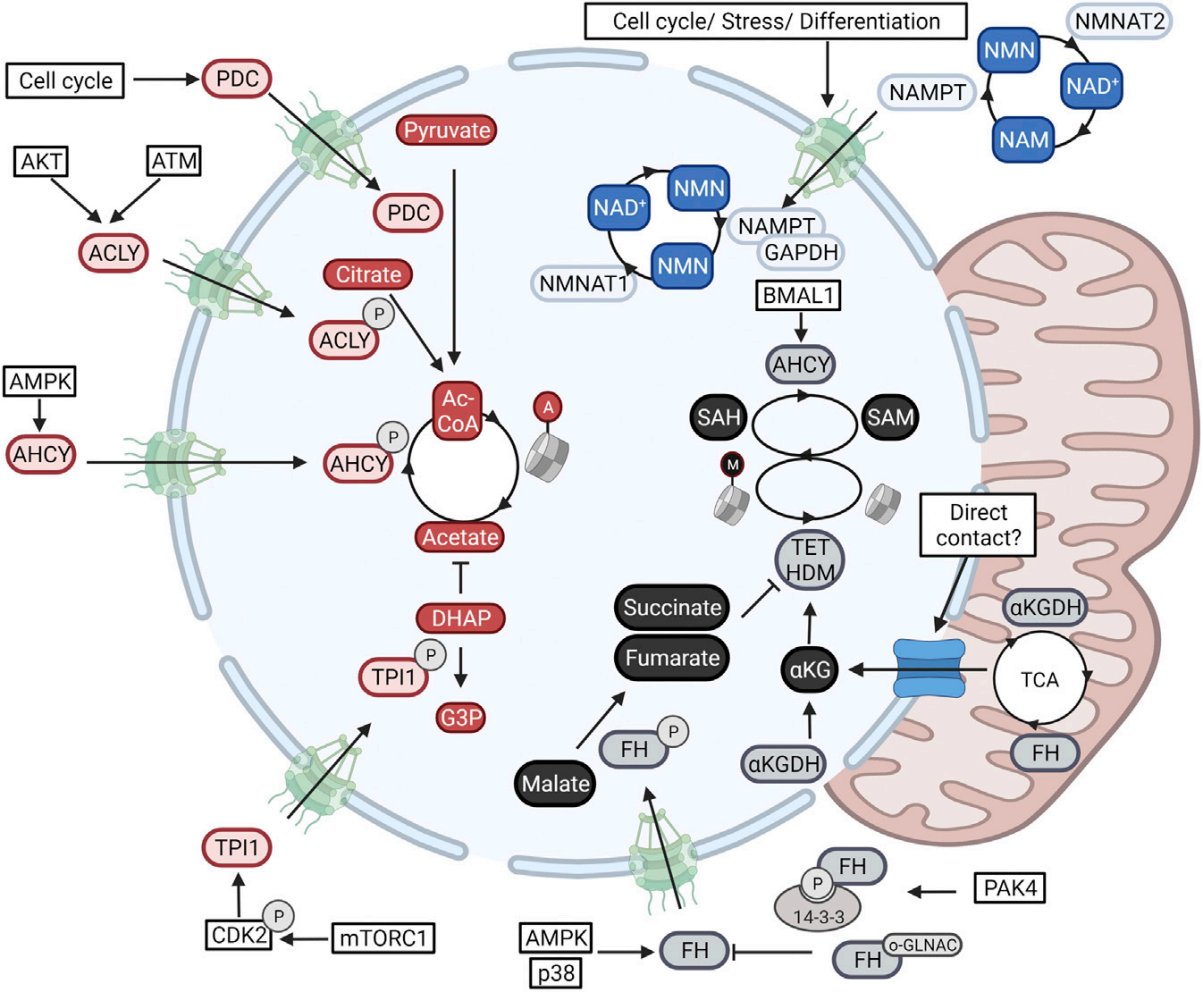
Nuclear metabolism is established through the in-and export of enzymes. Metabolic enzymes fueling NAD^+^-dependent deacetylation (Blue), acetyl-CoA-dependent acetylation (red), and SAM-dependent methylation (Black) are translocated to the nuclear compartment. Blue) Nuclear NAD^+^ cis modulated through the transcriptional control of the nuclear NAD^+^ producing enzyme NMNAT1 in with regards to those of NMNAT2 and NMNAT3 present in the cytoplasm and nucleus respectively. Second, the NMN-producing salvage enzyme NAMPT translocates to the nucleus downstream of signals involved in cellular stress and cell cycle. Red) Nuclear acetyl-CoA levels are determined through the nuclear import of the acetyl-CoA-producing enzymes ACLY, ACSS2, and PDC. Levels are finetuned even further through the import of the glycolytic enzyme TPI1 responsible for clearing the acetate scavenger DHAP. Black) Nuclear SAM and SAH levels are determined through the nuclear activity of MAT2A and AHCY respectively. Epigenetic demethylation is also regulated through nuclear levels of several TCA-cycle metabolites (αKG, succinate, and fumarate). These are modulated through the nuclear import of the αKG dehydrogenase complex (αKGDC), and FH. Additional studies have found direct contact points between the nucleus and mitochondria that can also account for metabolite exchange. Created with BioRender.com.

Metabolic compartmentalization is best illustrated with regards to NAD^+^ metabolism (Cambronne and Kraus, 2020). In the cytoplasm and nucleus, NAD^+^ is consumed by poly (adenosine diphosphate–ribose) polymerases (PARPs), sirtuins, and is used as a co-factor for biosynthetic reactions (Ryu et al., 2018). Importantly, here NAD^+^ acts as a rate-limiting resource capable of regulating reaction speeds. In the mitochondrial compartment, NAD^+^ cycles between a reduced and oxidized state to fuel ATP production. This much bigger pool of NAD^+^/NADH is physically separated from other pathways through the mitochondrial membrane and maintained through the selective import of NAD^+^ through its mitochondrial transporter (Davila et al., 2018). Although the nucleus and cytoplasm are not separated by a closed membrane, NAD^+^ levels do differ between both compartments (50–100 µ> in the cytoplasm, and 100–120 µM in the nucleus) (Sallin et al., 2018). As NAD^+^ is small enough to diffuse freely through the nuclear pore, this unexpected difference is due to local modulation of levels. Indeed, as illustrated in Figure 2, the enzymes responsible for generating and consuming NAD^+^ are compartmentalized and form a specific nuclear NAD^+^ metabolism (Boon et al., 2020a; Cambronne and Kraus, 2020).

NAD^+^ can be generated *de novo* from nicotinic acid (NA) or tryptophan or can be salvaged from NAD^+^ derived-degradation products. These pathways converge in the generation of nicotinic acid mononucleotide (NaMN) or nicotinamide mononucleotide (NMN) that are then condensed with AMP through the activity of compartmentalized NMN (NMNAT) enzymes (Cambronne and Kraus, 2020). Whereas the NMNAT1 isoform is located in the nucleus, NMNAT2 and NMNAT3 are directed to the cytoplasmic and mitochondrial compartments respectively (Berger et al., 2005). Knockout mice of any of the three enzymes are not viable indicating these enzymes to be non-redundant (Gilley and Coleman, 2010; Conforti et al., 2011; Hikosaka et al., 2014). Furthermore, axonal degeneration due to decreased NMNAT-2 activity can not be rescued by overexpression of the nuclear located NMNAT1 isoform (Conforti et al., 2007). In accordance, differentiation programs operating in pre-adipocytes induce a switch in expression from nuclear NMNAT1 to cytoplasmic NMNAT2. As both enzymes compete for NMN, a switch from nuclear to cytoplasmic production reduces nuclear NAD^+^ levels, leading to reduced PARP1-mediated ADP-ribosylation of pro-adipogenic C/EBPβ (Ryu et al., 2018).

Besides compartmentalized isoforms, NAD^+^ metabolism is tweaked further through the selective nuclear import and nuclear activation of the salvage enzyme NAMPT. NAMPT catalyzed the rate-limiting step in the regeneration of NAD^+^ after consumption by forming NMN from Niacinamide (NAM). A variety of signals including cell cycle progression, genotoxic or oxidative stress, and DNA damage, have been shown to increase nuclear translocation of NAMPT to boost nuclear NAD^+^ levels (Svoboda et al., 2019). Translocation is mediated by exposure of an endogenous nuclear localization domain (Svoboda et al., 2019), or through shuttling mediated by complexation with another metabolic enzyme; glyceraldehyde-3-phosphate dehydrogenase (GAPDH) (Grolla et al., 2020). Furthermore, within the nucleus, as a response to DNA damage and NAD^+^ availability, SIRT6 can activate both nuclear NAMPT (Sociali et al., 2019) and PARP1 (Mao et al., 2011) to balance nuclear NAD^+^ production with PARP1-mediated consumption. The compartmentalization of NAD^+^ metabolism thus clearly shows that the nucleus and cytoplasm are metabolically different. Nuclear metabolism forms a separate compartment through the local activity of metabolic enzymes.

## Nuclear Import of Metabolic Enzymes Determines Nuclear Metabolism

Nuclear metabolism goes beyond the nuclear production and consumption of NAD^+^. Histone acetylation is fueled by the activity of three protein complexes which all have been found to translocate to the nucleus under different conditions. Under energy stress, histone acetylation needs to be reorganized to drive the expression of an autophagic profile. As illustrated in Figure 2, under these conditions, AMPK induces the phosphorylation and subsequent import of ACSS2 for the nuclear recycling of HDAC-derived acetate for nuclear acetyl-CoA production. In this way, nuclear ACSS2 maintains the pools of nuclear acetyl-CoA during epigenetic reprogramming (Li et al., 2017). In accordance, a high need for nuclear acetyl-CoA during the DNA damage response was shown to induce nuclear import of ACLY through ATM- and Akt-dependent phosphorylation (Sivanand et al., 2017), whereas cell cycle progression is correlated with the sequential in-and export of the PDC (Sutendra et al., 2014). In addition, a recent study has found the nuclear activity of the glycolytic enzyme triosephosphate isomerase 1 (TPI1), to finetune histone acetylation further. A phosphorylation cascade composed of mTORC1 and CDK2 induces the phosphorylation and nuclear import of the enzyme to lower nuclear levels of its substrate dihydroxyacetone phosphate (DHAP). As DHAP is a scavenger of acetate, this allows for the accumulation of nuclear acetate levels which act as a fuel for nuclear acetyl-CoA production (Zhang et al., 2021) (Figure 2).

Histone and DNA methylation are also determined through the activity of nuclear metabolic enzymes. One single gene, methionine adenosyltransferase 2A (MAT2A, MAT1A is found in hepatocytes), converts the amino acid methionine into the universal methyl donor SAM. As shown in Figure 2, MAT2A acts both in the nucleus and cytoplasm and is needed to generate nuclear SAM for MT-dependent repression of MafK target genes (Katoh et al., 2011). Nuclear SAM metabolism is adjusted further through the nuclear import of the enzyme Adenosylhomocysteinase (AHCY). This enzyme, responsible for hydrolyzing SAH, the competitive inhibitor for SAM-dependent MTs, was found to cyclically operate in the nucleus and induce H3K4_me3_ downstream of the circadian clock (Greco et al., 2020).

Whereas MTs are regulated by nuclear levels of SAM and SAH, histone and DNA demethylation, catalyzed by lysine demethylase (LSD) (Shi et al., 2004), Jumonji C (JmjC) domain-containing proteins (Tsukada et al., 2006), and the ten-eleven translocation (TET) (Tahiliani et al., 2009) enzymes, is controlled by nuclear levels of TCA intermediated. Both the TET and JmjC- containing proteins are dioxygenases that are dependent on the availability of nuclear αKG and show competitive inhibition by αKG-related metabolites such as succinate, fumarate, and 2-hydroxyglutarate (2HG). Although these metabolites are generally regarded as part of the mitochondrial TCA cycle, a growing body of evidence has also identified their production in the nucleus. As shown in Figure 2, all mitochondrial enzymes responsible for the stepwise conversion of pyruvate to αKG were found to transiently operate in the nucleus of the developing zygote to sustain H3K4 and H3K27 acetylation and H3K27 trimethylation (Nagaraj et al., 2017). In this regard, mitochondria have been described to form contact points with the nuclear membrane through a translocator protein (TSP)O and protein kinase A (PKA)-dependent tethering, for the exchange of cholesterol and modulation of lipid content (Desai et al., 2020). Furthermore, currently unknown molecular signals can expose a nuclear localization signal in the αKG dehydrogenase (α-KGDH) complex and thus induce the nuclear production of succinyl-CoA for H3K79 succinylation through the acetyltransferase KAT2A (Wang et al., 2017a). In accordance, nuclear import of fumarate dehydrogenase was found essential for sustaining DNA repair. Indeed, nuclear fumarate inhibits KDM2B-mediated H3K36 demethylation, in turn enhancing the Ku70-mediated repair of DSBs (Jiang et al., 2015) (Figure 2).

Besides from metabolic enzymes fueling epigenetic methylation and acetylation, also other marks are now appreciated to be produced in the nucleus. As discussed before, histone lactylation might be driven by nuclear lactate dehydrogenase (LDH) (Ferriero et al., 2018). Additionally, under conditions of oxidative stress, LDHA non-canonically converts α-ketobutyrate (α-KB) to α-hydroxybutyrate (α-HB) specifically in the nucleus. This, in turn, leads to the activation of DOTL1, H3K79 hypermethylation, and induction of the anti-oxidant response in cervical tumors (Liu et al., 2018). Furthermore, several studies have shown Pyruvate kinase (PK) M2 to provide nuclear ATP for histone phosphorylation. This is induced by extracellular signal-regulated kinase (ERK)-mediated phosphorylation of S37 (Yang et al., 2012a) or p300- and JMJD5-mediated acetylation of K433 (Wang et al., 2014), both leading to the conversion of the PKM2 tetramer to a monomer, exposure of the NLS, and nuclear translocation. Clearly, the observation that so many metabolic enzymes are specifically targeted to the nuclear compartment indicates a nucleus-specific function. As elaborated on below, nuclear metabolism thus allows linking metabolic changes to specific epigenetic responses.

## Nuclear Metabolic and Epigenetic Enzymes Complexes on Chromatin

The selective import or exclusion of metabolic enzymes through the nuclear pore complex represents a mechanism to modulate the availability of metabolites in the nuclear compartment. Yet in most cases, a metabolically fueled epigenetic response should be tailored to specific genomic loci. Interestingly, nuclear metabolic enzymes are recruited onto chromatin and could therefore generate metabolites in subnuclear domains.

**FIGURE 3:**
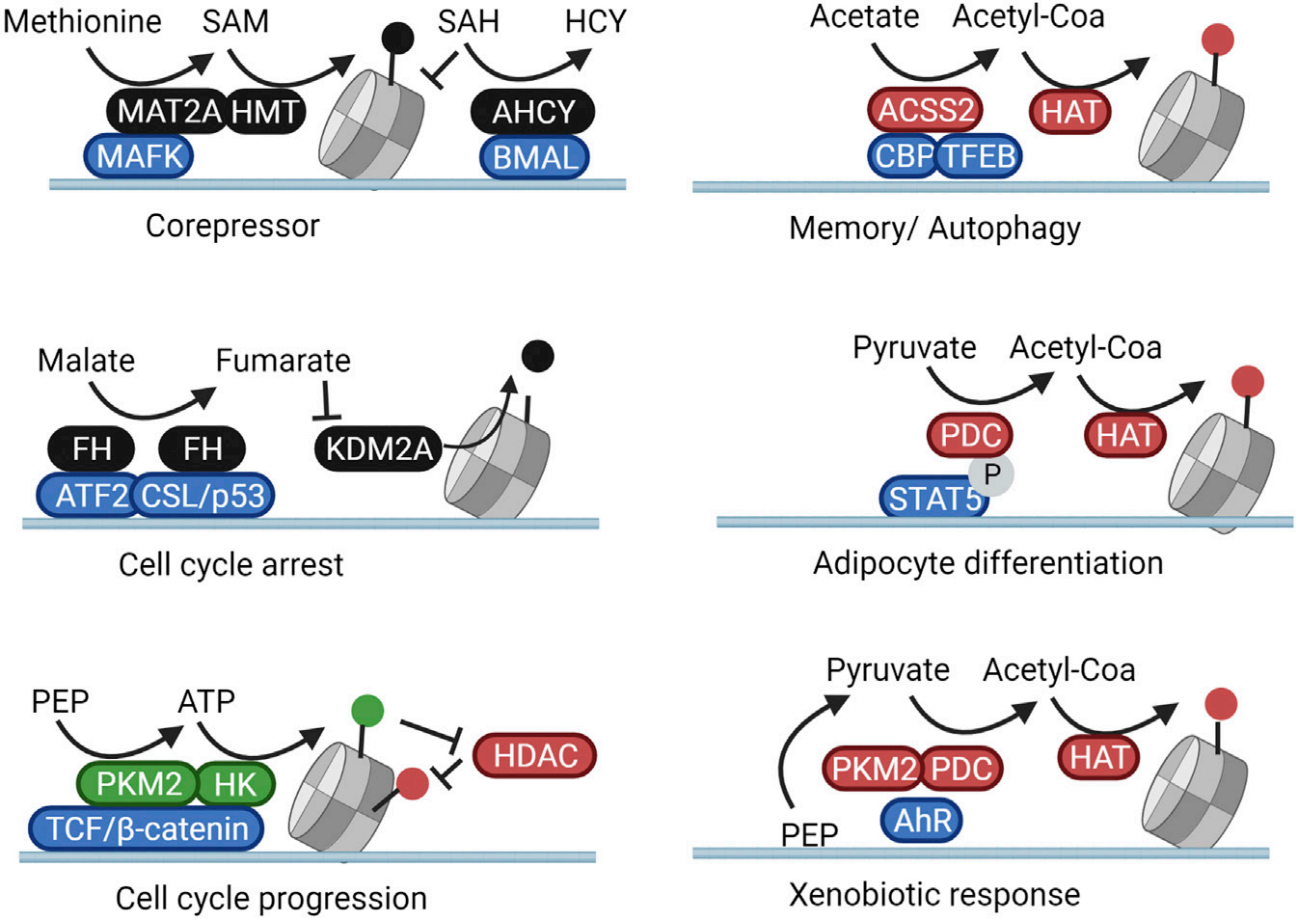
Subnuclear metabolism is directed towards loci through protein complexation. Nuclear metabolic enzymes are targeted to specific genetic loci through complexation with transcription factors. In these subnuclear compartments, they shape a local metabolic microenvironment that activates or inhibits the activity of resident chromatin modifiers. In this manner, metabolic changes result in specific cellular and phenotypical responses rather than general genome-wide reactions. Created with BioRender.com.

As previously described, MAT2A fuels HMT-dependent repression of Mafk target genes. As shown in Figure 3, this silencing occurs rather specifically as MAT2A itself physically interacts with MAFK and is targeted to the promoter regions of MAFK target genes to locally generate SAM (Katoh et al., 2011). In accordance, in the context of the circadian regulation of H3K4m^e3^, nuclear AHCY, responsible for removing the MAT2A inhibitor SAH, forms a complex with the circadian regulator Brain and Muscle ARNT-Like 1 (BMAL1). Cyclic recruitment of AHCY to circadian controlled genes is thus able to rhythmically derepress the production of SAM in these subnuclear compartments (Greco et al., 2020). Along the same lines, histone demethylation is also controlled locally through subnuclear fumarate synthesis through fumarate hydratase (FH). In pancreatic cancer cells, FH translocates to the nucleus and binds to the transcription factor ATF2 after AMPK-mediated translocation (pS75). The local production of fumarate from malate on ATF2-regulated promoters inhibits KDM2A and induces cell cycle arrest through elevation of H3K36me2 (Wang et al., 2017b). Similarly, p38-phosphorylated FH (pT90) downstream of TGFβ-signalling binds to the transcription factor CSL/p53 complex leading to an inhibition of H3K36 demethylation, enhanced p21 transcription, and cell growth arrest (Chen et al., 2019). The growth inhibitory activity of nuclear FH can be reversed through its cytosolic retention through PAK4-mediated phosphorylation at S46 (Chen et al., 2019) or through O-GlcNAcylation (Wang et al., 2017b) (Figures 2, 3).

Regarding the local regulation of histone acetylation, under deprivation conditions in brain tumors, nuclear-translocated ASCC2 binds to transcription factor EB (TFEB) and fuels histone acetylation from acetate specifically on the promoters of TFEB target genes responsible for lysosomal biogenesis and autophagy (Li et al., 2017). In accordance, in neurons of the hippocampus ACSS2 forms a complex with the acetyltransferase CBP and is thus recruited to the promoter of genes involved in memory consolidation (Mews et al., 2017). Interestingly, alcohol metabolism was also found to increase blood acetate levels, leading to a specific ACSS2-dependent deposition of acetylation marks regulating transcriptional programs linked to alcohol metabolization and addiction (Mews et al., 2019). In adipose tissue, nuclear PDC subunits are recruited to genes involved in adipocyte development through interaction with the phosphorylated form of the transcription factor STAT5 (Richard et al., 2017). In accordance, in the hepatic cell line HepG2, H3K9 acetylation of promoters involved in xenobiotic metabolism was regulated through the local activity of both PKM2 and the PDC, together responsible for shuttling glucose intermediates into nuclear acetyl-CoA pools. In this case, PKM2 and the PDC were directed to specific promoters by forming a complex with the transcription factor Aryl-hydrocarbon receptor (AhR) (Matsuda et al., 2016) (Figure 3).

The locus-specific activity of PKM2 has also been shown to locally modulate epigenetic phosphorylation. In a variety of cancer models, EGFR stimulation was found to induce phosphorylation and nuclear translocation of PKM2, which then binds to phosphorylated β-catenin. This complex allows for the generates of local ATP for the phosphorylation of H3 at T11 on β-catenin/TCF/LEF-downstream genes. H3 phosphorylation, in turn, inhibits binding of HDAC3, therefore increasing histone acetylation and driving the expression of cell-cyle and metabolic regulators such as cyclin D1 and c-Myc (Yang et al., 2011; Yang et al., 2012b). Interestingly, in yeast, enzymes responsible for the local production of ATP, SAMe, and acetyl-CoA (Pyk1, SAM synthetases, and an acetyl-CoA synthetase, respectively) were found to form a large chromatin-bound complex termed SESAME. This complex balances glucose availability with glycolytic flux through the combined modulation of SAM and ATP availability for H3K4 methylation and H3T11 phosphorylation on histones of the promoter of the yeast PKM2 homolog Pyk1 (Li et al., 2015). In a separate study, the acetyl-CoA generating subunit of SESAME, ACS2, was found to promote histone H4K16 acetylation (H4K16ac) enrichment at subtelomeric regions through direct complexation with the histone acetyltransferase SAS protein complex (Chen et al., 2021). Complexation of metabolic enzymes with transcription factors and epigenetic modifiers would thus integrate metabolism into the complex epigenetic machinery that shapes chromatin.

## Metabolic Enzymes Might Function in Phase-Separated Chromatin Microdomains

Metabolic enzymes that are recruited to genetic elements operate locally through metabolic-epigenetic complexes. However, it is unlikely that every promoter or genomic locus is occupied by a differential set of metabolic enzymes. As proposed by the late Paolo Sassone-Corsi, the trapping of metabolic enzymes in chromatin niches modulates the local metabolite concentrations of genomic microdomains (Katada et al., 2012). Indeed, despite possessing no internal membrane structures, the nucleus exhibits high levels of structural organization. Dozens of nuclear bodies (NBs), including the nucleolus, Cajal bodies, PML nuclear bodies, and Polycomb group bodies, form separated liquid droplets through phase separation. Transient protein-protein and protein-RNA interactions physically separate from the surroundings through a differential surface tension (Dundr, 2012; Sawyer et al., 2019). Although speculative, it is exciting to hypothesize that metabolic enzymes might also be included in these droplets and operate in small nanoreactors.

**FIGURE 4:**
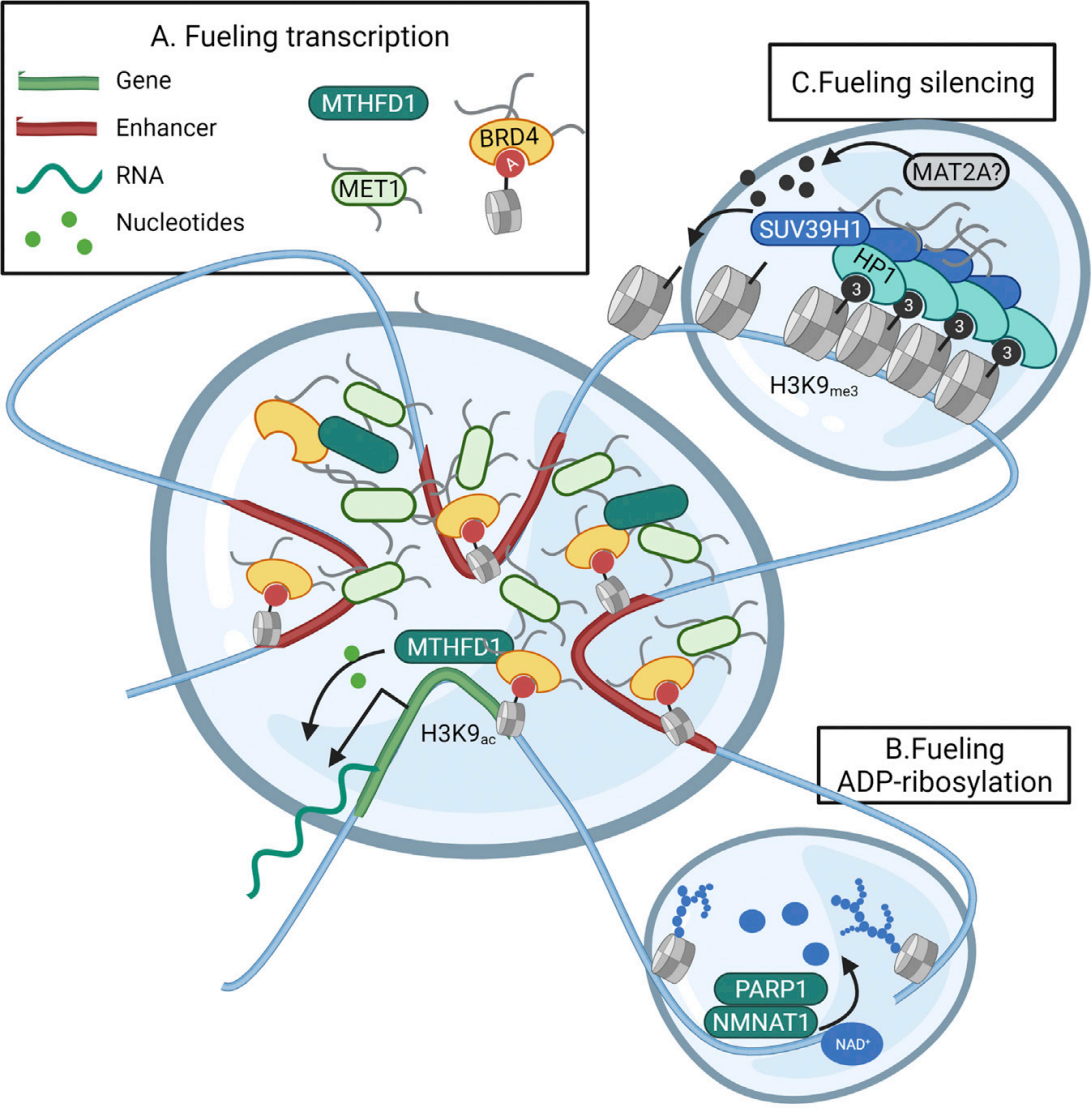
Subnuclear metabolism operates in liquid-liquid phase-separated domains. Nuclear metabolic enzymes might operate in liquid-liquid phase-separated droplets known to form the foundation of subnuclear compartmentalization. **(A)** Transcription is facilitated through the concentration of the transcriptional machinery, promoter regions, and super-enhancers in a separated droplet. This positional gathering is accomplished through interactions between transcription factors, and the intrinsically distorted regions (IDR) of the co-activators BRD4 and MED1. As the folate cycle enzyme MTHFD1 is recruited to the same location by BRD4, (parts of) nucleotide biosynthesis could fuel local nucleotide demand within the droplet. **(B)** During the initiation of the DNA repair response, liquid-liquid demixing occurs through the massive PARP-dependent ADP-ribosylation of histones and DNA-repair proteins. The PARP-interacting enzyme NMNAT1 could thus fuel the local need for NAD^+^ as a precursor for ADP-ribose within the droplet. **(C)** Silencing of heterochromatin occurs through phase-separation and compaction induced by HP1. HP1 also recruits the MT SUV39H1 responsible for spreading the H3K9me3 heterochromatin mark. It could be that this spreading requires the incorporation of the SAM-producing enzyme MAT2A within the heterochromatin droplet. Created with BioRender.com.

The need for metabolic activity in nuclear nanoreactors is clear with regards to fueling transcription in transcriptional hubs. The current model of transcriptional control consists of high concentrations of transcription factors that bind promoter and super-enhancers and cyclically bring these elements together in a phase-separated bubble that contains all proteins required for transcriptional bursts (Hnisz et al., 2017). As shown in embryonic stem cells, interactions between the pluripotency gene OCT4 and the intrinsically distorted regions (IDR) of the co-activators BRD4 and MED1 were found to form phase-separated condensates encompassing superenhancers (Sabari et al., 2018) and genes supporting the pluripotency network (Boija et al., 2018). In accordance, also the assembly of the super elongation complex responsible for transcriptional elongation requires the concentration of the SEC and the positive transcription elongation factor b (P-TEFb) in phase-separated condensates (Guo et al., 2020). Nucleotide-consuming transcription and DNA replication represent serious metabolic challenges for proliferating cells (Falkenberg et al., 2019; Hämäläinen et al., 2019). The metabolic enzyme Methylenetetrahydrofolate dehydrogenase (MTHFD) is responsible for generating the one-carbon units 10-formyltetrahydrofolate (10-formyl-THF) and 5,10-methylene tetrahydrofolate (5,10-methylene-THF) needed to build purines and pyrimidines. Especially the multienzyme complex responsible for catalysing the 10-step production of purines (purisome), has been found to form on the surface of mitochondria where the mitochondrial MTHFD2 variant provides immediate access to one-carbon units (Pareek et al., 2020). Yet, cytoplasmic MTHFD1 also translocates to the nuclear compartment when nuclear thymidine is low or under folate restriction (Field et al., 2016). Both thymidylate synthase (TS) and the pyrimidine-synthesising CAD multienzyme complex operate mainly in the nucleus (Sigoillot et al., 2005; Field et al., 2016). As a recent proteomics-based study also found the purine-synthesising PAICS, GART, ATIC, and ADSL enzymes on chromatin, this data suggests a nuclear-specific production of nucleotides (Sdelci et al., 2019). In addition, the same study showed acetylated nuclear MTHFD1 to be recruited by BRD4 to transcriptionally active regions. Indeed, DNA binding of the metabolic enzyme MTHFD1 was found to overlap with H3K27ac regions marking promoters and enhancers within the transcriptional hub (Sdelci et al., 2019). Furthermore, BRD4 is known to balance transcriptional activity with (local?) purine availability (Li et al., 2021). Although purine- and pyrimidine-synthesising enzymes were not directly linked with BRD4, this data does suggest a subnuclear complexation of metabolic enzymes to support transcriptional bursts (Figure 4A).

In accordance, the DNA damage response has been shown to rely on extensive binding of the DNA damage response effector 53BP1 around sites of breaks to selectively concentrate DNA response mediators (Kilic et al., 2019). Interestingly, the initial liquid-liquid demixing of the “DNA-response-condensate” from the nuclear environment is initiated by PARP1-mediated ADP-ribosylation of histones leading to decompaction of chromatin and recruitment of DNA repair factors (Altmeyer et al., 2015). As in MCF7 cells, the NAD^+^ consuming PARP1 has been found to physically interact with the NAD^+^ producing metabolic enzyme NMNAT1 to fuel ADP-ribosylation of local proteins, also in phase-separated condensates, NMNAT1 might fuel local PARP1 activity (Figure 4B) (Zhang et al., 2012). As a last possible example of metabolism operating in epigenetic liquid-liquid phase-separated droplets, one can look at the silencing of heterochromatin. The compaction of heterochromatin is accomplished through the spread of heterochromatin protein 1 (HP1) over large genomic areas. HP1 binds histones based on H3K9_me3_ and recruits the Histone-lysine N-methyltransferase SUV39H1 to methylate neighboring H3K9 and spread the methylation mark. HP1 also oligomerizes and forms such high local concentrations that non-interacting proteins are excluded from the genomic region, thus forming a separated lipid droplet (Larson et al., 2017). Since the interacting SUV39H1 requires SAM availability for methylating H3K9, it would be interesting to hypothesize MAT2A to be recruited within the HP-1 droplets rather than relying on passive diffusion of nuclear SAM (Figure 4C).

Despite few examples known within the nuclear compartment, it is very well established that metabolic enzymes for phase-separated condensates (Prouteau and Loewith, 2018). In yeast, hypoxic stress induces the Snf1p-dependent assembly of glycolytic enzymes in membraneless g bodies to increase glycolytic flux (Jin et al., 2017). Notably, the glycolytic metabolon can also become sequestered to the cytoskeleton during endothelial tip formation to locally generate ATP (De Bock et al., 2013; Sweetlove and Fernie, 2018). These examples clearly show the value of metabolic complex formation in cellular sublocations. It remains to be seen whether metabolic complexes indeed also lie at the heart of epigenetic condensates, and whether mechanisms exist to control the import or exclusion of metabolic pathways within these droplets.

## Concluding Remarks

The field of epigenomics has evolved tremendously from the first descriptions of DNA methylation changes during developing embryos. Although the image of epigenetic marks as static guardians of cellular identity as culminated in Waddington’s epigenomic landscape is, of course, valid, the epigenome also represents a much more dynamic manner of regulating transcription (Allis and Jenuwein, 2016). As epigenetic modifiers are enzymes, they can probe the cellular metabolome and sense changes in levels of metabolites and vitamins. As described, this mechanism allows for balancing metabolite levels through rapid transcriptional changes. Inputs into the transcriptional regulation through the metabolic epigenome include nutritional status (Boon et al., 2020a) (including oxygen availability Thienpont et al., 2016 and pH McBrian et al., 2013), signaling pathways (Carrer et al., 2019), circadian oscillations (Masri and Sassone-Corsi, 2013; Greco et al., 2020).

Yet, this interaction can also be the cause of disease. For example, a reduction in TET activity in hypoxic tumors leads to tumor evolution through the hypermethylation of tumor suppressors (Thienpont et al., 2016). Furthermore, methionine starvation within tumors inhibits the deposition of H3K79_me2_, resulting in low expression of STAT5 and immune dysfunction (Bian et al., 2020). Nevertheless, metabolic pathways also represent ideal targets to treat disease and epigenetic dysfunction. Dietary interventions or modulation of the intestinal microbiota can lead to a better outcome when combined with additional therapy (Iida et al., 2013; Gao et al., 2019; Parkhitko et al., 2019; Zheng et al., 2020).

To fully take advantage of metabolism as a therapeutical target, it is of extreme importance to understand how the interconnection between metabolism and epigenetics is regulated on a molecular level. Metabolic interventions lead to changes in epigenetic mark deposition. Furthermore, given that the Km values of epigenetic modifiers sometimes differ by several orders of magnitude, the intrinsic activity of chromatin modifiers might explain the specificity of marker deposition in different metabolic states (Reid et al., 2017). Yet, the numerous examples of nuclear-translocated metabolic enzymes demonstrate that molecular mechanisms exist to translate cytoplasmic cues into a nuclear metabolism. Especially for metabolic transformations, location matters and epigenetic modifiers probably respond more to local fluctuations in metabolite levels than those observed in other organelles Future studies should thus be focused on understanding subcellular metabolism and metabolic compartmentalization. This would encompass uncovering the regulation of nuclear translocation and investigating the subnuclear activity of metabolic enzymes and their operation in phase-separated liquid-liquid droplets. Interestingly, the activity and stability of transcription factors, generally regarded as the master regulators of expression, are also highly influenced by post-translational modifications (Benayoun and Veitia, 2009). The possibility thus arises that, as for the epigenome, also the local activity of transcription factors is determined through resident metabolic enzymes. Metabolic enzymes determine cell fate and transcription through epigenetic and transcription factor modification. Yet, it remains to be seen whether the location of metabolic enzymes on DNA matters. The key in understanding the interaction between metabolism and epigenetic modifications thus lies in studying their location.

## Data Availability

The original contributions presented in the study are included in the article/supplementary material, further inquiries can be directed to the corresponding author.
